# AquaCrop modeling for sustainable potato irrigation: trade-offs between yield and crop water productivity

**DOI:** 10.3389/fpls.2025.1624099

**Published:** 2025-08-11

**Authors:** Abraham Rai, Nawab Ali, Younsuk Dong

**Affiliations:** Biosystems and Agricultural Engineering, Michigan State University, East Lansing, MI, United States

**Keywords:** AquaCrop, potato, yield, crop water productivity, irrigation scenarios, trade-off analysis

## Abstract

Potato is an important staple food crop for global food security, and its productivity is sensitive to water availability, making precision irrigation management crucial for optimum yield and crop water productivity ( $WP_{C}$ ). The AquaCrop model was calibrated and validated under 50% and 70% field capacity (FC) managed through SoilWatch 10 moisture sensors at depths of 15, 30, and 45 cm. Thereafter, different irrigation scenarios, from 20% to 90% FC, were developed and simulated across 10 years (2014–2024), classified into wet (>312.9 mm), normal (256–312.9 mm), and dry (<256 mm) years based on the total crop growing season rainfall for yield and $WP_{C}$ across two soil types. Trade-off analyses were performed for all scenarios across all years—normal, wet, and dry years—to assess the relationship between yield and $WP_{C}$ , simulating yield effectively with an index of agreement (IA) of 0.999. The results indicated that the simulated yield at harvest closely matched the observed yield (±10%), suggesting significant accuracy of the model. The soil water content (SWC) estimations under both treatments were satisfactory, with the IA and the Nash–Sutcliffe model efficiency coefficient (NSE) both close to 1. Scenario analysis exhibited variations in the yield and $WP_{C}$ for irrigation treatments across soil types. Trade-off analysis showed that irrigation at 40%–60% FC resulted in better yield and $WP_{C}$ , as categorized in the win–win scenarios, across all years and two different soil characteristics. Similarly, the correlation analysis revealed that the mid-tuber to the late-tuber bulk stages were critical for irrigation supplementation, corroborated by the findings of the 40%–70% FC irrigation scenarios. As such, AquaCrop could be a feasible modeling tool to optimize irrigation for potato yield and $WP_{C}$ under climate variability.

## 1 Introduction

Potato production plays an important role in contributing to food security for the increasing global population. Potato production is surpassed in worldwide production only by three other major crops—maize, wheat, and rice—and is the leading vegetable crop consumed in the United States. According to the Food and Agricultural Organization (FAO) 2023 statistics, potato is cultivated and harvested in an area of approximately 17 million hectares, with global production totaling 383 million tonnes (FAO, 2023). The United States ranks among the top producers globally for potato production, contributing to domestic consumption and international trade (Knudson and Miller, 2023). In the 2023 growing season, potato was grown and harvested on an area of 961,100 acres, an increase of 5% from the 2022 growing season, with the total production amounting to 440 million cwt (hundredweight), an increase of 9% from the 2022 growing season. The total value of potatoes sold in 2023 was US $5 billion, an increase of 3% from the previous year, with the number of potatoes sold accounting for 92% of the total 2023 production (USDA-NASS, 2024).

Climate change has been a significant challenge for the agriculture sector, causing the onset of variable temperatures and unpredictable and inconsistent rainfall. Potato production is vulnerable to climate changes, such as drought events and heat anomalies, which are impacted by the shallow root system of the crop (Thornton, 2020). Potato production depends on water management (rainfall and irrigation), soil management practices, seed quality, fertilizer application, soil moisture, elevation, and slope. The developmental stages of potato (i.e., sprouting, emergence, tuber formation, and bulking) are sensitive to temperature (Levy, 1986). Climate changes affect the potato phenology, especially causing advancement or delay in leaf emergence and dropping, tuber initiation bulking, and maturity based on the location (Alva, 2008). Approximately 400–800 mm of water is required for successful potato production, which invariably relies on meteorological factors and other variables (Badr et al., 2012; Cantore et al., 2014). Decreasing the water from 60% to 65% results in drought conditions that affect the growth rate, while excessive water application causes leaching and tissue decay, i.e., blackheart (Levy, 1986; Stark et al., 2020). Therefore, irrigation management plays a vital role in potato production, significantly influencing yield and quality. Potatoes exhibit a shallow root system with a higher water demand, particularly during the tuber formation and the bulking stages. A consistent irrigation supply helps maintain the soil moisture and maximize the tuber yield, size, and quality. Water stress conditions or the lack of irrigation results in smaller tubers, lower yield, and higher susceptibility to diseases (Begum et al., 2018; King et al., 2020). Efficient irrigation management practices not only maintain the soil moisture but also mitigate the leaching of nutrients. Optimum irrigation management during the critical stages of the crop is essential for maintaining healthy crops with better yield and quality. The irrigation application methods, the irrigation regimes, and the irrigation application times are crucial in potato production, directly impacting the yield, quality, and $WP_{C}$ (Dong et al., 2023). As such, a well-planned irrigation schedule implementation that matches the crop water needs at critical stages is essential to avoid water stress, reduce water loss and leaching nutrients, and to maintain optimum production and quality (Sieczka et al., 1992). The global water usage for potatoes accounts for approximately 287 m^−3^ tons, in light of the 126 crops researched over a frame of a decade (Mekonnen and Hoekstra, 2011). The challenges posed by climate change, in terms of unpredictable rainfall and drought conditions, in turn increase the agricultural water demands, making it pertinent to optimize the $WP_{C}$ . Hence, the integration of simulation models is a strategy to optimize irrigation management, resulting in enhanced crop yields and $WP_{C}$ (Paredes et al., 2018).

Previously, several simulation models have been used to assess the productivity and $WP_{C}$ of potato under diverse climatic conditions. Potato modeling was initiated in 1980 (MacKerron and Waister, 1985), and since then, approximately 30 different models have been developed. Some notable examples include LINTUL-POTATO (Kooman and Haverkort, 1995), CropSyst (Stöckle et al., 2003), SIMPOTATO (Hodges et al., 1992), SUBSTOR-Potato (IBSNAT, 1993), MOPECO (Martínez-Romero et al., 2019), SIMDualKc (Paredes et al., 2018), and AquaCrop (Patel et al., 2008). Of these models, AquaCrop stands out for its simplicity, accuracy, and robustness, making it the preferred choice for many researchers (Patel et al., 2008; Farahani et al., 2009; Abedinpour et al., 2012; García-Vila and Fereres, 2012; Katerji et al., 2013; Montoya et al., 2016; Yin et al., 2023). AquaCrop is a water-driven, process-based model that simulates biomass and yield. It is used for decision-making, planning, and scenario analysis under various irrigation schedules and diverse field–climatic conditions (Shirazi et al., 2021). In comparison to various other prevalent models, AquaCrop has advantages as it has an easy-to-use interface and is highly accurate and intuitive in simulating the yield and $WP_{C}$ under different agronomic practices (Abedinpour et al., 2012). Previous studies that used AquaCrop on potato production have explored estimation of yields under different irrigation methods, such as drip and furrow irrigation (Wale et al., 2022; Izadi et al., 2023), and varying climatic conditions (Casa et al., 2013; Ahmadi et al., 2022). However, the existing literature focused on short-term assessments in dry climates, while long-term scenario analyses for sustainable potato production under humid climates using AquaCrop are limited. To address this gap, the AquaCrop model was implemented to study the effect of multiple irrigation regimes under sprinkler irrigation on the yield and $WP_{C}$ under humid climatic conditions for long-term scenario analysis to optimize irrigation management for potato production.

## 2 Materials and methods

### 2.1 Experimental site description

The experiment was conducted at Montcalm Research Center, Michigan, USA (43°21′14.76” N, 85°10′44.76” W, at an elevation of 290 m above sea level), and a commercial farm (Mecosta, Michigan, USA) during the 2023 potato growing season. The climate in the study area is classified as humid, with an average annual rainfall of 821.25 mm and an average annual evapotranspiration of 730.0 mm. Soil samples were collected at three soil depths (15, 30, and 45 cm) and were tested for composition, bulk density, and volumetric water content (*θ*_v_) by the Michigan State University Soil Laboratory (East Lansing, MI, USA). The characteristics of the soils for each field experimental site are shown in **Table 1**.

**Table 1:** Soil properties of the experimental fields at the Montcalm and Mecosta sites.

Site	Soil depth (cm)	Bulk density (g cm^−3^)	Sand (%)	Silt (%)	Clay (%)	Soil texture
Montcalm	0–15	1.471	74.5	13.8	11.7	Sandy loam
	15–30	1.255	73.5	14.8	11.7	Sandy loam
	30–45	1.369	78.5	10.8	10.7	Sandy loam
Mecosta	0–15	1.564	80.5	9.90	9.60	Loamy sand
	15–30	1.578	80.5	9.40	10.1	Loamy sand
	30–45	1.600	83.5	7.90	8.60	Loamy sand

### 2.2 Experimental design

The effect of irrigation regime on the potato yield and $WP_{C}$ in humid climate was assessed with an experiment conducted in a randomized complete block design (RCBD) split-plot arrangement with three replications. The experiment comprised irrigation treatments of 50% and 70% of the field capacity (FC), as represented in **Table 2**. Irrigation was applied through an overhead irrigation system to irrigate at different FC thresholds. Prior to the experiment, an irrigation distribution uniformity evaluation was performed to ensure that the irrigated plots in each field receive the same amount of irrigation water.

**Table 2:** Treatments and irrigation application details for the experimental site.

Treatment	Planting date	Start of irrigation	Harvest date	No. of irrigations	Irrigation amount (mm)
50% FC	May 10	June 04	September 22	3	48
70% FC		May 31		5	80

FC, field capacity.

### 2.3 Field data collection

The LOCOMOS (Low-Cost Sensor Monitoring System), an Internet of Things (IoT)-based system, was installed in-field to monitor the soil moisture and the environmental variables (Dong et al., 2024). SoilWatch 10 sensors by Pino-Tech (Stargard, Poland) were used to monitor the soil moisture levels at depths of 15, 30, and 45 cm in the soil profile. The collected data were then sent to the LOCOMOS IoT web cloud server every 15 min, as well as being saved in an SD card as backup. LOCOMOS utilized a 12-V 7A battery, a 12-V solar panel, and solar battery charging controllers to power the system. The amount of irrigation applied for each treatment during the crop growing season was tracked manually.

The growth of potatoes was monitored weekly to determine the development stages based on visual observations. A middle row, 3.05 m in length, for each replication was used for the measurement of tuber yield, with the harvested tubers placed in bags for yield determination. The harvested tubers were sorted into different tuber sizes based on classes PO, B, A1, A2, and OV1 according to the United States Department of Agriculture potato grading and classification system (USDA, 2011). The harvested tubers for each replication were also subsampled for scab rating to verify their quality.

### 2.4 Crop water productivity

The efficiency of irrigation in crop production is significantly influenced by the amount of water applied relative to the needs of the crops and the uniformity of its application. $WP_{C}$ is one of the most utilized parameters describing the effectiveness of irrigation in terms of crop yield. The $WP_{C}$ was determined as in Equation 1 (Rodrigues and Pereira, 2009; Fernández et al., 2020).


(1) $$WP_{C}\;=\;\frac{Yield\;}{TWU}$$

where $WP_{C}$ is the crop water productivity (in kilograms per cubic meter); Yield is the economic yield (in kilograms per hectare); and TWU is the total amount of water (in cubic meter per hectare) used in crop production.

### 2.5 AquaCrop model description

**Figure 1:**
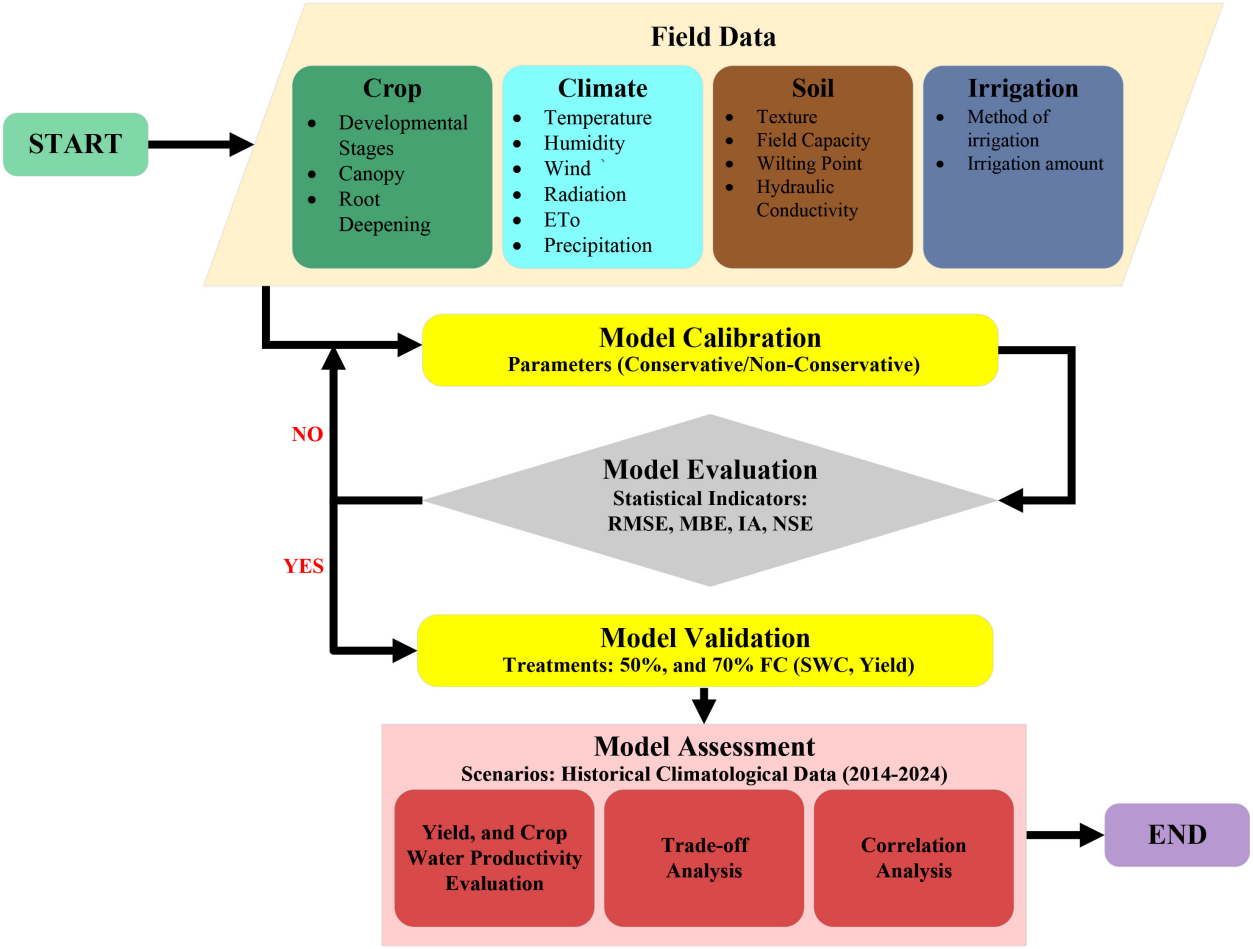
Process flowchart of the AquaCrop model for the simulation of potato yield and $WP_{C}$ .

AquaCrop is the water productivity model developed by the Land and Water Division of the United Nations, Food and Agriculture Organization (FAO). The AquaCrop model (version 7.1) was used to optimize irrigation management for yield prediction under climate change scenarios. **Figure 1** shows the processes for the AquaCrop model calibration, validation, and simulation of potato yield and $WP_{C}$ . The model simulates crop growth and biomass progression based on the soil water and salt balance, the atmospheric parameters (i.e., temperature, rainfall, ET_0_, and atmospheric CO_2_ concentration), the crop characteristics (e.g., water productivity and crop coefficient, among others), and the field characteristics (e.g., irrigation schedule, the type of irrigation system implemented, soil profile, etc.) (Steduto et al., 2009; Montoya et al., 2016).

The model simulates the water use and the yield of the crop based on the categorization of the actual evapotranspiration (ET) into soil evaporation (E) and crop transpiration (Tr), as well as the final yield (*Y*) into biomass (*B*) and harvest index (HI), as expressed in Equation 2 (Raes et al., 2016).


(2) ET=E+Tr

where E is a function of the evaporation reduction coefficient (Kr) , the soil evaporation coefficient (Ke) , and the reference evaporation rate ${\text{(ET}}_{o})$ , which is an index for the evaporation to the atmosphere. Tr is dependent on the soil water stress coefficient (Ks) , the cold stress coefficient ${\text{(Ks}}_{Tr})$ , and the crop transpiration coefficient ${\text{(Kc}}_{Tr})$ proportional to the green canopy cover of the crop (Raes et al., 2016). The final yield for the crop was simulated based on the HI and the aboveground biomass production, as expressed in Equation 3.


(3) $$\text{Y }={\text{f}}_{HI}*{\text{HI}}_{o}.\text{B}$$

where ${\text{f}}_{HI}$ is a multiplier that considers the stresses that adjust the HI from its reference value, which takes into account the effects of stressors–water, temperature–at the instance of yield formation and crop pollination; ${\text{HI}}_{o}$ is the reference harvest index; and *B* is the aboveground biomass production (Raes et al., 2016).

The SWC in the soil profile was determined using a soil water balance approach, which takes into account the incoming (rain, irrigation, and capillary rise) and outgoing (surface runoff, deep percolation, evaporation, and crop transpiration) water fluxes. Rainfall and the irrigation events were user-defined inputs, while the other components of the soil water balance were computed based on simulations of the canopy development, the depth of the groundwater table, and the soil characteristics (Van Gaelen, 2016). At a particular soil volume, the water content was expressed as an equivalent depth, making it convenient to track the incoming and outgoing water fluxes. The stored soil water in the root zone, expressed as a depth, was simulated based on Equation 4.


(4) $${\text{W}}_{r}=1000\;\theta {\text{ Z}}_{r}\;\left(1-\;\frac{\text{Vol}{\%}_{gravel}}{100}\right)$$

where ${\text{W}}_{r}$ is the SWC of the root zone expressed as depth (in millimeters); θ is the average volumetric water content in the fine soil fraction of the root zone (in cubic meters per cubic meter); ${\text{Z}}_{r}$ is the effective rooting depth; and $\text{Vol}{\%}_{gravel}$ is the volume percentage of the gravel fraction in the root zone (Raes et al., 2016).

### 2.6 Input data for the AquaCrop model

#### 2.6.1 Soil characteristics

The data input with regard to the soil characteristics for the AquaCrop model consisted of the soil profile categorized into three different depths, with properties such as texture, volumetric water content, and bulk density, as shown in **Table 1**. The soil data were imported into the model to create a soil data file. For the groundwater section of the soil parameter, the depth of groundwater at the experimental site was 9.14 m, with the depth being considered a supplementary water source for crop growth.

#### 2.6.2 Weather data

**Figure 2:**
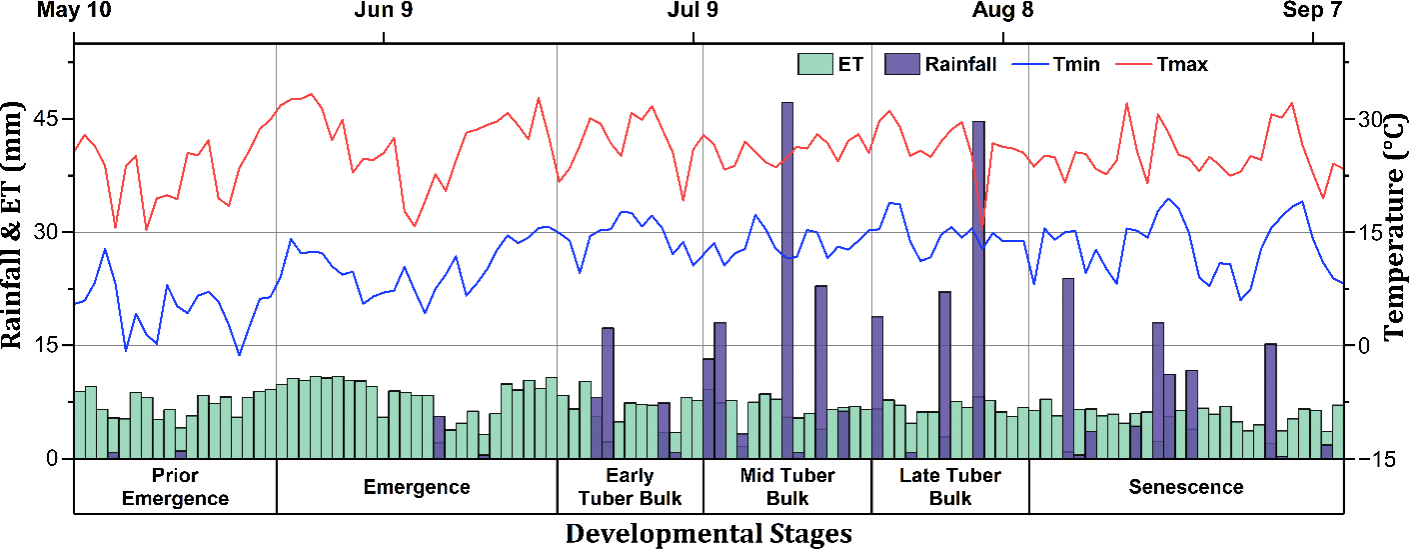
Daily maximum and minimum temperature, $ET_{o}$ , and rainfall of the experimental site for the 2023 growing season.

The weather data were obtained from Michigan State University Enviroweather (https://enviroweather.msu.edu/), which consisted of rainfall (in millimeters), maximum and minimum relative humidity (in percentage), maximum and minimum temperature (in degree Celsius), wind speed (in meters per second, at 3 m), and solar radiation (megajoules per square meter). The daily reference evapotranspiration ( $ET_{o})$ was calculated using the FAO Penman–Monteith method (Allen et al., 1998). **Figure 2** shows the daily maximum and minimum atmospheric temperature, rainfall, and ETo  for the 2023 growing season. The total rainfall during the growing period was 312.8 mm, with the highest amount of rainfall, i.e., 111.7 mm, occurring during the mid-tuber bulk stage of potato production. Moreover, the long-term historical meteorological data, 2014–2024, utilized for the scenario analysis were also obtained from the Enviroweather station.

#### 2.6.3 Crop characteristics

The data for the crop characteristics, including the developmental stages, crop evapotranspiration, production, and the water–temperature–fertility stressors, were used in the AquaCrop model. The initial canopy cover, canopy development, flower yield formation, senescence, and root deepening factors of the crop data were based on visual field observations, with the crop development shown in **Table 3**. The water productivity, HI, moisture, and temperature stress response coefficients were determined based on the baseline parameters provided by the model, with adjustments made using the trial-and-error method, further elaborated in *Section 3.1.1*. The atmospheric CO_2_ concentration utilized in the model simulation was based on the default data recommended by the model, MaunaLoa.CO_2_.

**Table 3:** Crop critical developmental stage initiation and termination expressed in days after planting (DAP) for the 2023 growing season.

Critical stage	Initiation		Termination	
	Date	DAP	Date	DAP
Planting	May 10	–	–	–
Emergence	May 28	21	–	–
Tuber initiation	June 22	43	July 05	56
Early-tuber bulk	July 05	56	July 20	71
Mid-tuber bulk	July 20	71	August 03	85
Late-tuber bulk	August 03	85	August 17	99
Vine kill	August 30	112	–	–
Harvest date	September 22	135	–	–

### 2.7 Model evaluation

The AquaCrop model was validated based on statistical evaluation metrics by assessing the measured and the predicted parameters. The root mean square error (RMSE) was utilized to assess the magnitude of prediction error (Equation 5). The mean bias error (MBE) was used to identify biased prediction and to determine the underestimation or the overestimation by the model (Equation 6). For estimation of the agreement between the observed and the predicted values, the IA was used (Equation 7). The Nash–Sutcliffe model efficiency coefficient (NSE) was used to determine model fitness to the data (Equation 8).


(5) $$RMSE=\sqrt{\frac{1}{N}\sum_{i=1}^{N}{\left(M_{i}-P_{i}\right)}^{2}}$$

(6) $$MBE=\frac{1}{N}\sum_{i=1}^{N}\left(P_{i}-M_{i}\right)$$

(7) $$IA=1-\frac{\frac{1}{N}\sum_{i=1}^{N}{\left(M_{i}-P_{i}\right)}^{2}}{\sum_{i=1}^{N}{\left(\left|P_{i}-\bar{M}\right|+\left|M_{i}-\bar{M}\right|\right)}^{2}}$$

(8) $$NSE=1-\frac{\sum_{i=1}^{N}{\left(M_{i}-P_{i}\right)}^{2}}{\sum_{i=1}^{N}{\left(M_{i}-\bar{M}\right)}^{2}}$$

where *N* indicates the sample size; *M* is the measured value; *P* refers to the observed value from the model; and *M¯* refers to the averaged measured value. The units of RMSE and MBE coincided with the units of the parameters involved. IA and NSE are dimensionless, with ranges from 0 (no agreement) to 1 (perfect match), indicating that the higher the value, the better the agreement between the measured and the estimated values.

### 2.8 Scenario analysis

**Figure 3:**
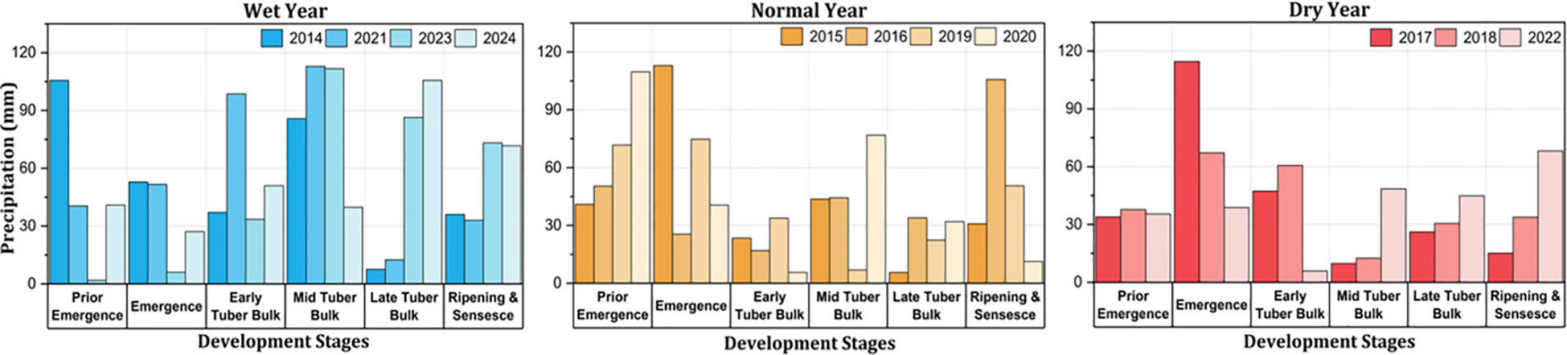
Categorization of the years based on the potato growing season rainfall: wet years (>312.9 mm), normal years (256–312.9 mm), and dry years (<256 mm).

The seasonal rainfall during the 2014–2024 potato growing seasons showed considerable variability across developmental stages, as illustrated in **Figure 3**. Higher rainfall typically occurred during the emergence, the mid-tuber bulk, and the late-tuber bulk stages, while the early-tuber bulk and senescence stages received lower rainfall. Notable peaks were observed in 2014 and 2017 before emergence; in 2023, during the mid-tuber bulk stage. In contrast, the lowest rainfall was recorded in the late-tuber bulk stage during the year 2023, a stage exhibiting the higher interannual variability. In order to account for the year-to-year variability in the total seasonal rainfall, the study period (2014–2024) was classified into wet, normal, and dry categories. This categorization was based on the percent deviation from the long-term annual rainfall (284.5 mm) using a ±10% threshold following methodologies based on reports from the Intergovernmental Panel on Climate Change (IPCC, 2007; Lobell and Field, 2007). The years exceeding +10% of the mean (>312.9 mm) were categorized as wet (2014, 2021, 2023, and 2024), those below −10% (<256 mm) as dry (2017, 2018, and 2022), and the rest as normal (2015, 2016, 2019, and 2020).

**Figure 4:**
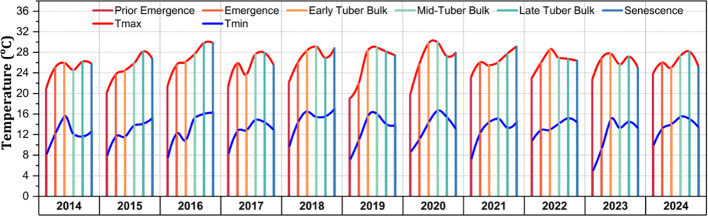
Maximum and minimum temperatures (in degree Celsius) of the experimental site based on the developmental stages for the 2014–2024 crop growing seasons.

The changes in the average maximum and minimum temperatures across the developmental stages for the study period (2014–2024) are shown in **Figure 4**. In general, the maximum temperature ( Tmax ) values revealed a cyclic pattern, with annual peaks aligning with the late-tuber bulk stage, with the average Tmax across the study period being 25.7°C. Similarly, the minimum temperature ( Tmin ) exhibited a trend of gradual increases over time, with the average Tmin across the study period being 12.9°C. Tmin showed lower variability, but a general upward trend, revealing potential climate change patterns. The temperature fluctuations were most notable in the growing seasons 2017, 2020, and 2023, depicting warmer conditions. Among the developmental stages, the Tmax was notably variable during the late-tuber bulk stage, while the Tmin remained more stable.

### 2.9 Statistical and trade-off analyses

The statistical performance indicators RMSE, MBE, IA, and NSE, expressed mathematically in *Section 2.9*, were used to comprehensively assess the accuracy and reliability of the model. The trade-off analysis of all the irrigation scenarios between the yield and $WP_{C}$ was based on the log response ratio (LnRR) calculated to measure the effect size (Hedges et al., 1999). The LnRR between yield and $WP_{C}$ for all treatments was calculated using Equation 9.


(9) $$lnRR\;=ln\;\left(\frac{xi}{xc}\right)=lnxi-lnxc$$

where xi refers to the treatment mean and xc indicates the control treatment for yield and $WP_{C}$ . The normally practiced irrigation, i.e., 50% FC, was considered as the standard treatment (control) for all the years, with the rainfed irrigation representing the growing conditions solely under rainfall, i.e., devoid of irrigation events. The calculated effect size values for yield and $WP_{C}$ were plotted on two-dimensional scatter plots to assess the trade-off and synergies across the treatments. The LnRR yield and the LnRR $WP_{C}$ were kept on the *X*- and the *Y*-axis, respectively. The plot was divided into four quadrants comprising the win–win (+, +), lose–lose (−, −), win–lose (+, −), and lose–win (−, +) scenarios based on *x* = 0 and *y* = 0. This visualization enabled a clear trade-off between yield and $WP_{C}$ . Furthermore, the scenario years categorized as wet, normal, and dry years (*Section 2.8*) were visualized separately for the yield and $WP_{C}$ trade-off.

The correlation between the irrigation regimes and their effects on yield, according to the developmental stages, was assessed based on the Pearson’s correlation coefficient (*r*), expressed in Equation 10. The degree of correlation between attributes can vary from “−1,” indicating inversely related, to “+1,” indicating a high similarity, with “0” indicating uncorrelated attributes (Pearson, 1895).


(10) $$r=\frac{n\left(\sum \;xy\right)\;-\;\left(\sum \;x\right)\left(\sum \;y\right)}{\sqrt{n\sum \;x^{2}\;-\;{\left(\sum \;x\right)}^{2}}\sqrt{n\sum \;y^{2}\;-\;{\left(\sum \;y\right)}^{2}}}$$

where *x* and *y* represent individual sample points and *n* represents the sample size. The data analysis and visualization were conducted using the software Origin (Pro) 2024b, version 10.15 (OriginLab Corporation, Northampton, MA, USA).

## 3 Results and discussion

### 3.1 Performance of the AquaCrop model

#### 3.1.1 Model calibration

The biomass progression simulation of the AquaCrop model takes into account the atmospheric parameters (temperature, rainfall, ETo , and CO_2_ concentration), the crop characteristics (crop coefficient, HI, and water productivity), and the field management parameters (irrigation method and irrigation scheduling), in addition to the soil water balance. The parameters involved in the simulation were categorized as either non-conservative or conservative. Conservative parameters are specific to a particular crop and remain consistent with time, location/climate, and management practices (Hsiao et al., 2009; Raes et al., 2009; Steduto et al., 2009). The model calibration was conducted through an adaptive process, with consideration of the appropriate values that closely simulated the crop parameters, in particular the SWC and crop yield, with the implemented values obtained from several sources: experimental data from the 2023 growing season, the AquaCrop reference manual (Raes et al., 2012), and other conducted studies (Montoya et al., 2016; Yin et al., 2023).

The parameters that were calibrated included canopy cover, crop development, effective root depth, and water productivity, in addition to the HI. The parameters for the calibrated AquaCrop model are as specified in **Table 4**. The model was considered to be well calibrated when the simulated and the measured values for SWC had RMSE and IA values closer to 0 and 1, respectively (Raes et al., 2012; Montoya et al., 2016; Yin et al., 2023). Furthermore, yield simulations were considered to be acceptable when the differences between the measured and the simulated values were within ±10% (Farahani et al., 2009). The results demonstrated agreement between the simulated and the observed values, indicating that the calibrated model parameters are reliable for the simulation of the growth and development of crops under the study scenarios.

**Table 4:** Parameters with calibrated values of the AquaCrop model.

Parameter		Calibrated value	Unit
Crop development and yield formation			
Initial canopy cover	CC_0_	0.6	%
Maximum canopy cover	CC*_x_*	92.0	%
Crop growth coefficient	CGC	1.80	% day^−1^
Crop decay coefficient	CDC	0.80	% day^−1^
Base temperature	*T*_base_	2.0	°C
Upper limit temperature	*T*_upper_	26.0	°C
Maximum effective root depth	–	0.47	m
Crop transpiration coefficient	$Kc_{Tr}$	1.10	–
Reference harvest index	$HI_{0}$	75.0	%
Water productivity	WP	19.0	g m^−2^
Water extraction pattern	–	40–30–20–10	%
Soil water stress			
Upper threshold for canopy expansion	$P_{exp,\;\;upper}$	0.26	–
Lower threshold for canopy expansion	$P_{exp,\;\;lower}$	0.66	–
Shape factor for canopy expansion	–	3.0	–
Upper threshold for stomatal closure	$P_{clo,\;\;upper}$	0.65	–
Shape factor for stomatal closure	–	3.0	–
Upper threshold for early canopy senescence	$P_{exp,\;\;upper}$	0.69	–
Shape factor for early canopy senescence	–	3.0	–

#### 3.1.2 Soil water content comparison

**Figure 5:**
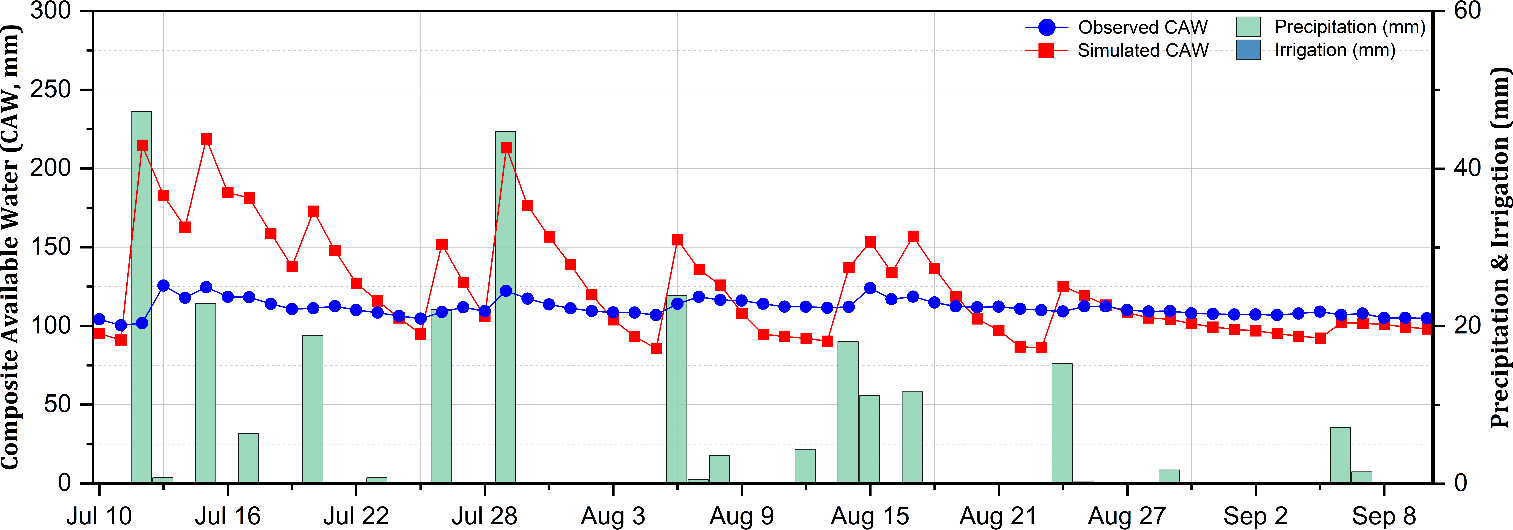
Observed and simulated composite available water (CAW; in millimeters) in the soil during the potato growing season under the 50% field capacity (FC) irrigation treatment.

**Figure 6:**
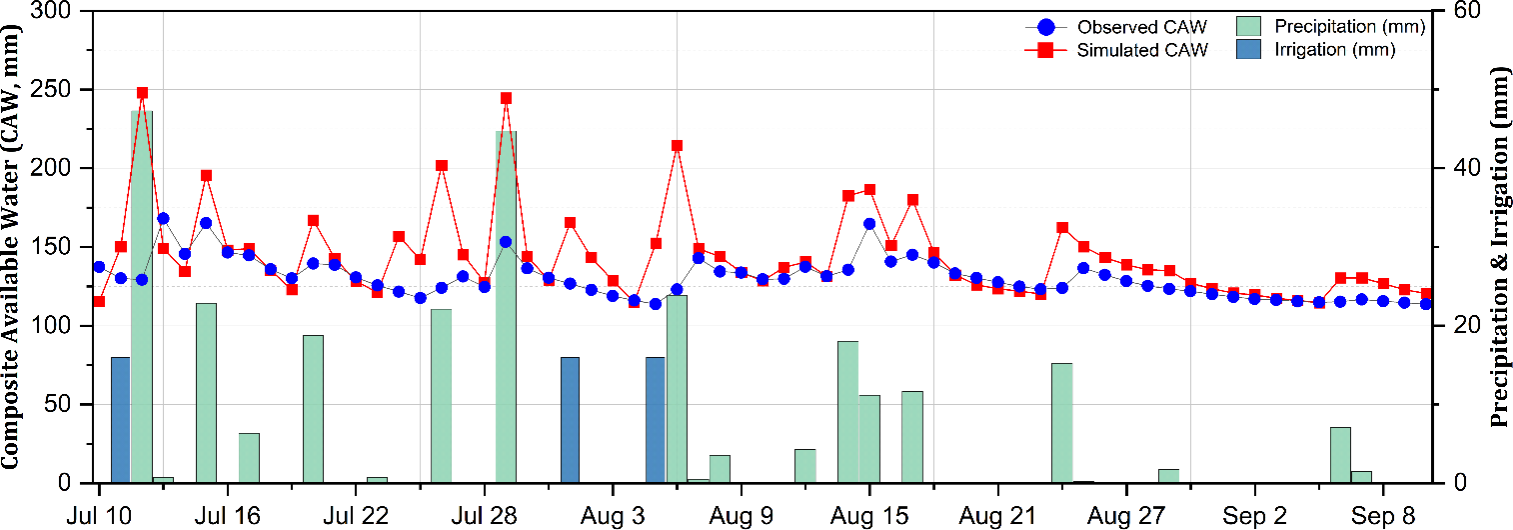
Observed and simulated composite available water (CAW; in millimeters) in the soil during the potato growing season under the 70% field capacity (FC) irrigation treatment.

**Figures 5**, **6** illustrate the temporal dynamics of the SWC in relation to climate and the irrigation characteristics at the experimental site throughout the 2023 growing season, depicting the SWC changes under 50% and 70% irrigation levels, respectively, based on the FC when utilizing the calibrated parameters.

**Figure 7:**
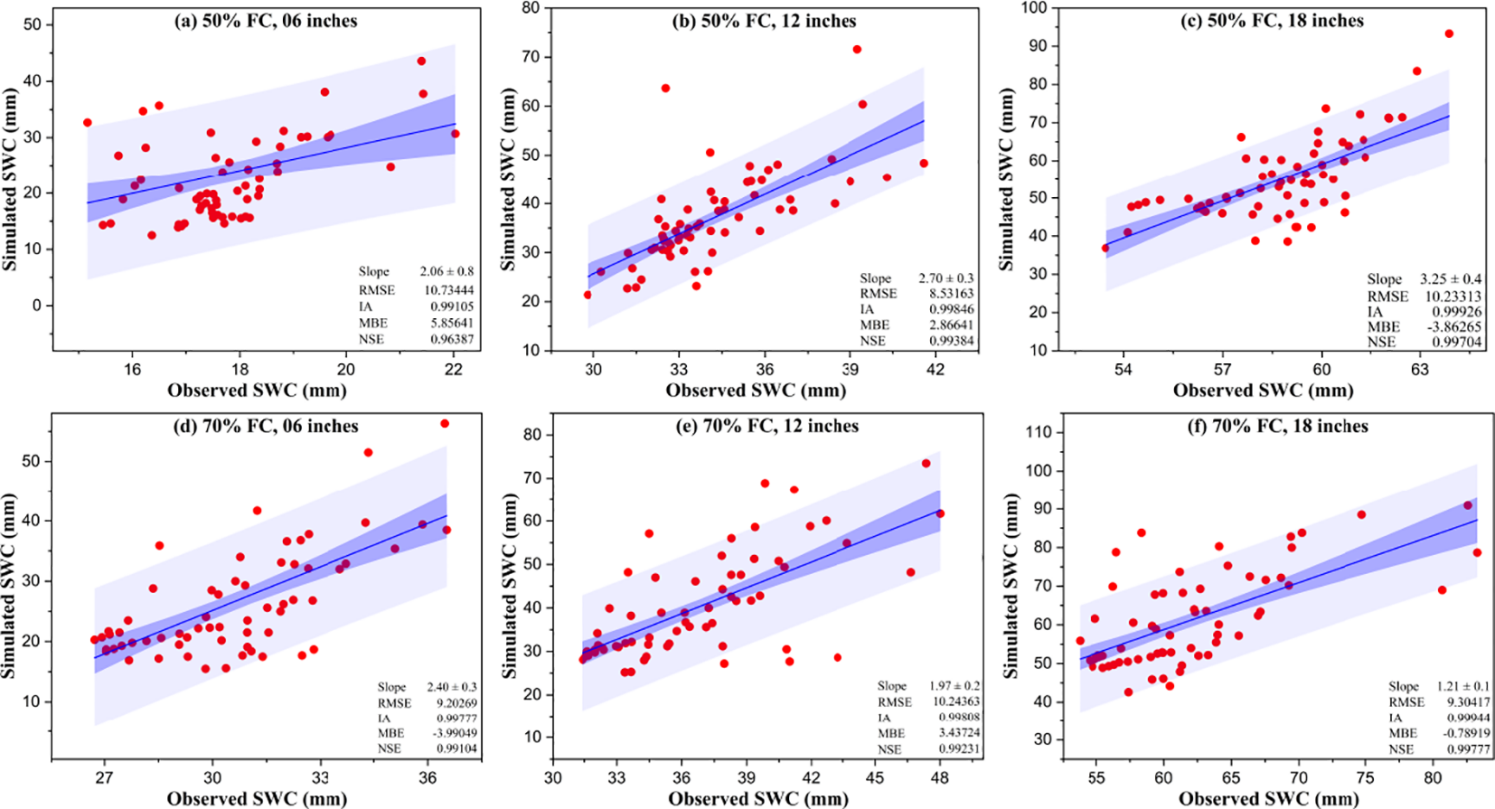
Comparison of the observed *vs*. the simulated soil water content (SWC) values of 50% field capacity (FC) **(a–c)** and 70% FC **(d–f)** for the specified observation soil depths with the respective evaluation parameters.

A trend of overestimation was found particularly in the 50% FC treatment. The estimation errors, the IA, and the model efficiency in relation to the simulation of the SWC under different irrigation levels, as well as sensor depths, in terms of the calibrated parameters are presented in **Table 5**. The estimation errors with the AquaCrop simulations were relatively low, with the RMSE ranging from 9.20 to 12.89 mm, corresponding to a variation of 8.1%–11.3% of the total available soil water. Furthermore, the MBE confirmed the overestimation trend for the 50% FC treatment, particularly at shallower depths, while under the 70% FC treatment, the MBE indicated a mix of over- and underestimation trends. The IA and NSE of the simulated results revealed satisfactory estimations with values closer to 1, indicating strong model performance despite localized biases (Zhang et al., 2022). These results align with previous studies of the AquaCrop model conducted on potatoes (Montoya et al., 2016; Paredes et al., 2018) and other crops (Katerji et al., 2013; Paredes et al., 2014). Operating the model under default parameters as outlined in the AquaCrop manual (Raes et al., 2012), the results exhibited a clear trend of overestimation, with the RMSE ranging from 9.56 to 24.18 mm, corresponding to 9.6%–24.2% of the total available water. This overestimation in the simulated SWC after model calibration is potentially due to inaccuracies in the estimation of the soil evaporation (Equation 1) and the crop transpiration (Equation 2). Previous studies revealed a similar pattern where the crop transpiration tends to be overestimated while the soil evaporation is often underestimated, contributing to biases in the estimation of the SWC. One potential reason for this discrepancy is that the crop transpiration coefficient ( $Kc_{Tr}$ ) of the model remains unaffected by water stress, unless the canopy development curve itself is impacted (Paredes et al., 2018). The values obtained from the calibration of the model evaluation parameters in terms of the observed and the simulated SWC values are illustrated in **Figure 7**.

**Table 5:** Statistical performance metrics of the model evaluation for the soil water content.

Treatment	Depth (cm)	RMSE (mm)	MBE (mm)	IA	NSE
50% FC	15	10.734	5.8564	0.991	0.964
	30	8.532	2.8664	0.998	0.994
	45	10.233	−3.862	0.999	0.997
70% FC	15	9.203	−3.990	0.998	0.991
	30	10.244	3.437	0.998	0.992
	45	9.304	−0.789	0.999	0.998

FC, field capacity.

#### 3.1.3 Yield comparison

The observed and the simulated crop yields for the 2023 growing season, along with the estimation error and the IA of the model, are presented in **Table 6**. The observed yield represents the average of 12 field replications, while the simulated yield represents a single simulated value of the AquaCrop model for each irrigation treatment. This is because the crop characteristics, along with the climate data, remain consistent across all treatments, with the only variable factor being the number of irrigation events applied for each treatment. The simulated yield values at harvest closely matched the observed field values, with differences within ±10% across treatments, which aligns with previous findings on the accuracy of the model (Farahani et al., 2009; Montoya et al., 2016; Yin et al., 2023). The estimation errors were relatively low, with RMSE values of 0.37 ton ha^−1^ for 50% FC and 2.65 ton ha^−1^ for 70% FC, along with the IA values indicating excellent agreement between the observed and the simulated values for yield. In terms of $WP_{C}$ , the 50% FC treatment demonstrated higher efficiency compared with 70% FC, reflecting greater water savings due to fewer irrigation events. Hence, the results demonstrated the significant accuracy of the AquaCrop model in simulating yield across varying irrigation scenarios, providing a theoretical framework for potato production studies across varied simulation scenarios.

**Table 6:** Statistical performance metric for model evaluation of potato yield.

Treatment	Observed yield (ton ha^−1^)	Simulated yield (ton ha^−1^)	RMSE (ton ha^−1^)	MBE (ton ha^−1^)	IA	NSE
50% FC	38.547	38.923	0.376	0.375	1.000	1.000
70% FC	45.495	41.323	2.657	−2.656	0.999	0.999

RMSE, root mean square error; MBE, mean bias error; IA, index of agreement; NSE, Nash–Sutcliffe model efficiency coefficient; FC, field capacity.

### 3.2 Changes in the yield and crop water productivity based on the irrigation thresholds

**Figure 8:**
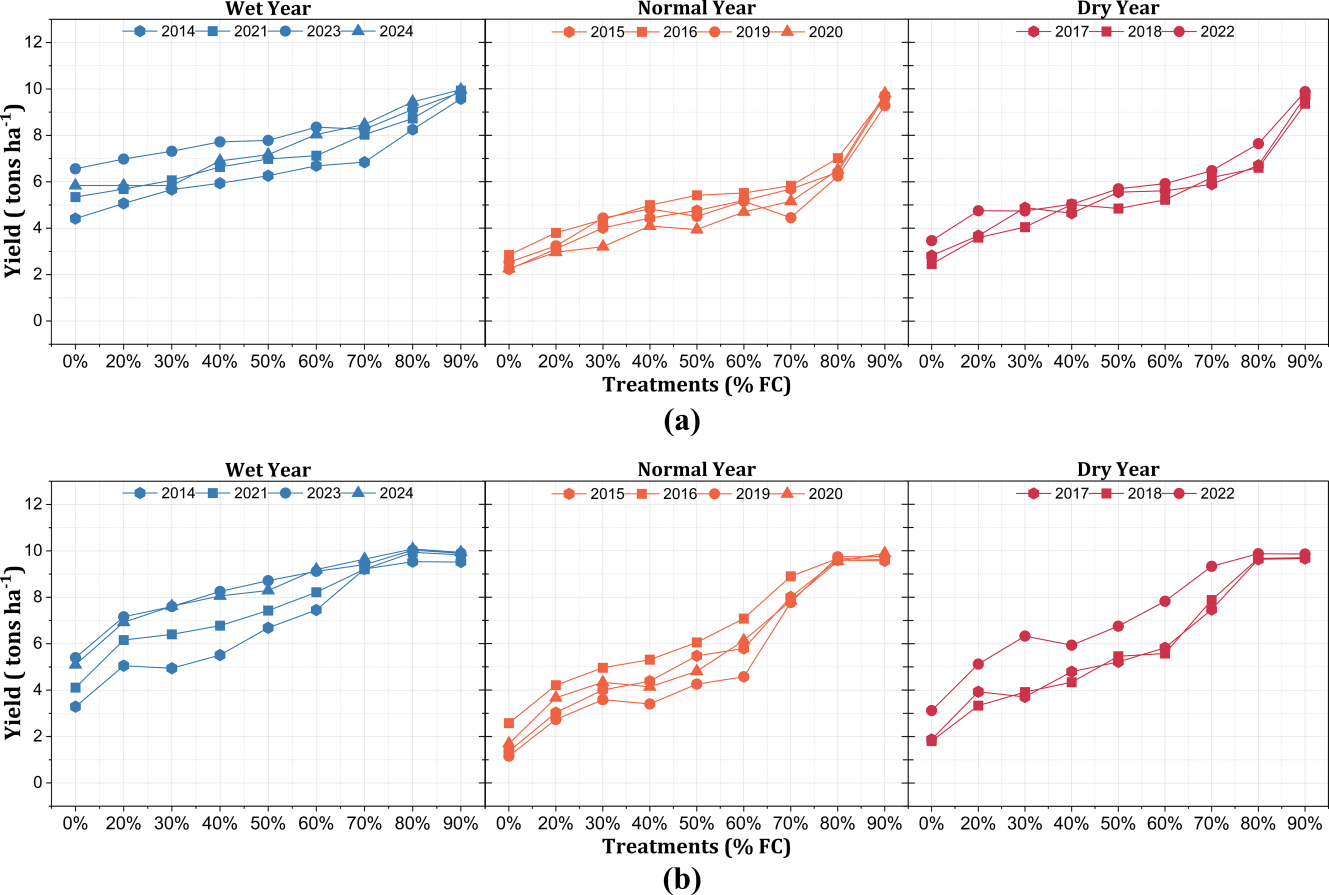
Potato yield (in tons per hectare) under different irrigation treatments for the Montcalm **(a)** and Mecosta **(b)** locations across 10 years (2014–2024), categorized into wet years, normal years, and dry years based on the rainfall for the growing season.

Scenario analysis was conducted for the study period 2014–2024 in order to evaluate the effects of varying irrigation thresholds on the yield and $WP_{C}$ under field conditions. The simulations were conducted for two distinct locations—Montcalm and Mecosta—with irrigation treatments ranging from 20% to 90% FC. The irrigation scheduling was based on IrrigMSU, a custom-built irrigation application developed by the Irrigation Labs, Michigan State University, with water application of 15.24 mm per irrigation event and the number of irrigation events dependent on the climatic data, the soil type, the crop developmental stage, and the irrigation threshold. The yield simulations revealed distinct trends across soil textures and rainfall categories (**Figure 8**). For Montcalm, which has a sandy loam soil, the yield consistently increased with higher irrigation treatments, particularly in the dry years, with a sharp increase in yield observed between 80% and 90% FC, coinciding with the near doubling of irrigation events (from 13 to 26 per season). However, in wet years, 70% FC was optimal and could potentially be reduced to 60% FC, without requiring additional irrigation. In normal years, 70% FC ensured sufficient yield results without excessive irrigation application, while a reduction in the irrigation events below 70% posed a mild risk to the yield results. For Mecosta, which has a loamy sand soil, the yield peaked at 80% FC, beyond which additional irrigation resulted in diminished returns. The higher infiltration rate of loamy sand resulted in excess irrigation beyond 80% FC not significantly increasing the yield, particularly in wet years. In normal and dry years, the optimal yield occurred at 80% FC, with irrigation events ranging from 26 to 29; however, exceeding 80% FC provided minimal yield benefits. The distribution of irrigation events over the growing season suggests that the irrigation events were concentrated during the critical growth phases of the crops (i.e., from the early-tuber to the late-tuber bulk stage), where the water demand was highest, particularly in dry years.

**Figure 9:**
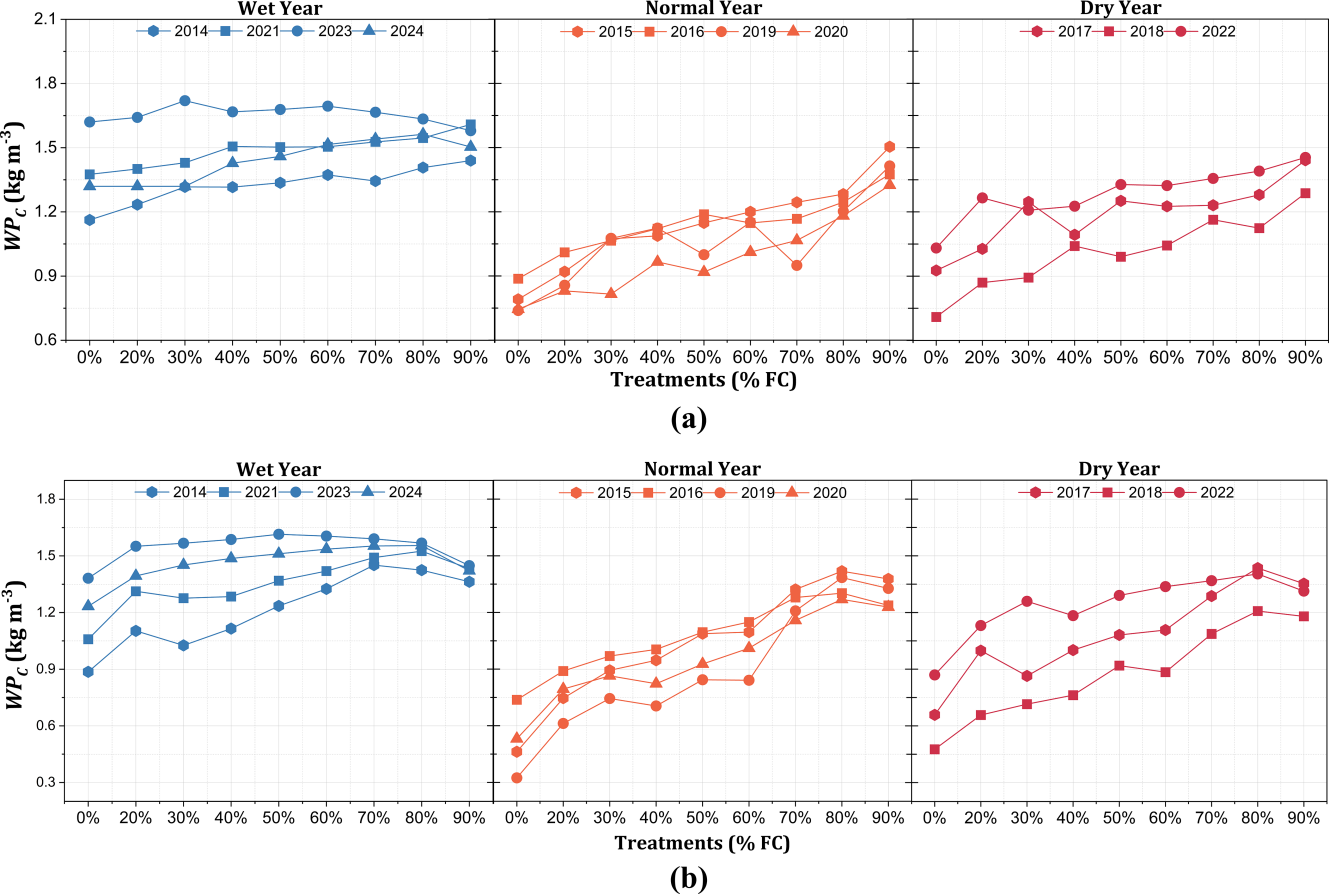
Crop water productivity ( $WP_{C}$ , in kilograms per cubic meter) of potato under different irrigation treatments in the Montcalm **(a)** and Mecosta **(b)** locations across 10 years (2014–2024) categorized into wet years, normal years, and dry years based on the rainfall for the growing season.

The $WP_{C}$ simulations demonstrated varying responses to the irrigation treatments across different conditions (**Figure 9**). For Montcalm, the $WP_{C}$ remained relatively stable in wet years, but declined beyond 70% FC, suggesting that excessive irrigation led to reduced efficiency. In normal years, the $WP_{C}$ exhibited a strong increasing trend, with a sharp increase from 80% to 90% FC, aligning with the increased instances of irrigation events. However, in dry years, 80% FC was necessary to maintain $WP_{C}$ . Beyond 80% FC increases in $WP_{C}$ values were less noticeable, suggesting that, while higher irrigation is necessary in dry conditions, excessive water use leads to inefficiencies. In contrast, for Mecosta, the $WP_{C}$ peaked at 80% in both normal and dry years, beyond which further irrigation (exceeding 50 irrigation events at 90% FC) did not improve efficiency. In wet years, 70% FC was sufficient to sustain $WP_{C}$ , with additional irrigation being unnecessary. The data on the frequency of irrigation events in Mecosta revealed that excessive irrigation resulted in more frequent applications across the season; however, the $WP_{C}$ gains were limited beyond 80% FC, particularly in the dry years.

#### 3.2.1 Trade-off analysis

**Figure 10:**
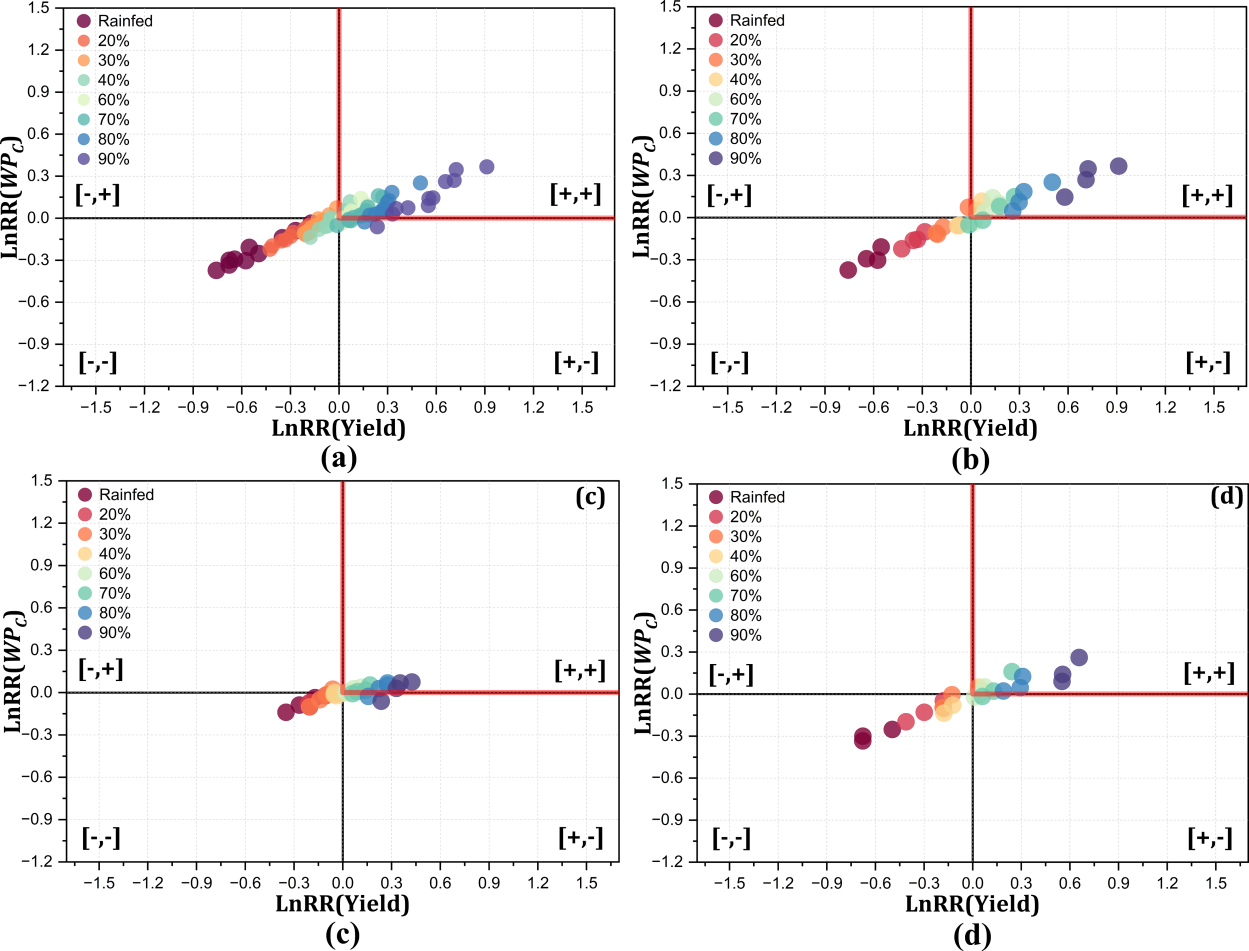
Trade-off analysis between yield and $WP_{C}$ for Montcalm (sandy loam) under all irrigation treatments for the 10 years **(a)**, normal years **(b)**, wet years **(c)**, and dry years **(d)** for the 2014–2024 crop growing seasons.

The trade-off analysis between crop yield and $WP_{C}$ offered critical insights into optimizing the irrigation management practices under diverse climatic conditions. For assessment of the climate variability impact, scenarios for yield and $WP_{C}$ were developed with the calibrated model from the 2014–2024 growing seasons. A quadrant-based approach was employed separately for all scenario years (2014–2024), including normal, wet, and dry years. For clear interpretations, trade-offs were employed separately for each category in different windows for all irrigation treatments (20%–90% FC, including rainfed). **Figure 10** shows the yield and $WP_{C}$ trade-off across scenario years for the Montcalm location with different magnitudes and fluctuations of yield and $WP_{C}$ for all irrigation treatments. **Figure 10a** displays the deficit irrigation treatments (20% and 30% FC and rainfed) falling into the lose–lose quadrant, the moderate irrigation treatments (40% and 60% FC) showing a win–win scenario for yield and $WP_{C}$ , and higher irrigation treatments (>60% FC) lying in the win–win and win–lose quadrants with comparable yield and $WP_{C}$ to moderate irrigation treatments. During normal years with 256–312.9 mm rainfall, the trade-off showed almost similar responses for the overall scenario years (**Figure 10b**). $WP_{C}$ tended to decrease during the wet years for higher irrigation treatments, and the majority of the trade-off that occurred in the lose–lose quadrant for deficit treatments and moderate irrigation treatments also showed reduction under wet years (**Figure 10c**). Compared with the wet years, the irrigation treatments in the dry years showed a distinct trend, with deficit irrigation treatments (20% and 30% FC and rainfed) in the lose–lose quadrant and moderate treatments (40% and 60% FC) in the win–win quadrant (**Figure 10d**). For the Montcalm location with sandy loam soil, the yield was decreased by 5%–27% for irrigation lower than 40% FC, with a subsequent decrease in $WP_{C}$ by 2%–12% across all the years. Higher irrigation scenarios (>60% FC) were shown to increase both the yield and $WP_{C}$ through the model; however, these excessive irrigation applications affected the crop growth and yield adversely. These findings align with Bani-Hani et al. (2018), who stated that moderate irrigation enhances the $WP_{C}$ and yield. Deficit irrigation than the optimum severely affects the potato yield (Waqas et al., 2021). Akkamis and Caliskan (2023) stated that excessive irrigation application decreases the potato yield. Furthermore, soil type has great influence on the $WP_{C}$ and yield, as reported by Hatfield et al. (2001).

**Figure 11:**
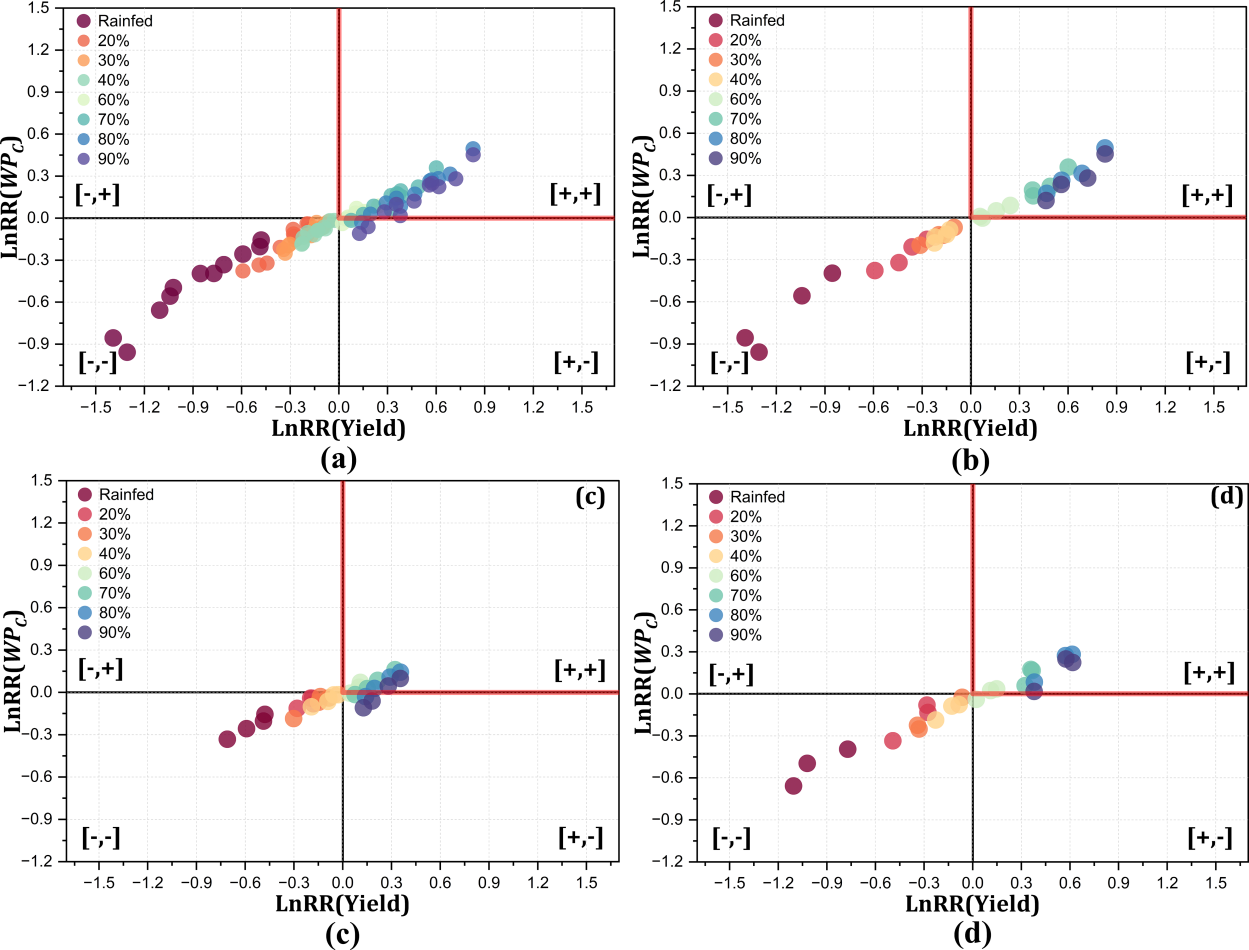
Trade-off analysis between yield and $WP_{C}$ for Mecosta (loamy sand) under all irrigation treatments for all years **(a)**, normal years **(b)**, wet years **(c)**, and dry years **(d)** for the 2014–2024 crop growing seasons.

The trade-off between yield and $WP_{C}$ for the Mecosta location differed from that of Motcalm, with large variations among the irrigation treatments due to the loamy sand soil type, as represented in **Figure 11**. The overall trend of trade-off analysis between yield and $WP_{C}$ revealed 20%, 30%, and 40% FC, and rainfed treatments fell in the lose-lose scenarios, while the higher irrigation treatment (>60% FC) showed a win–win scenario, although the 80% and 90% FC treatments were comparable to 60% FC in terms of yield and $WP_{C}$ . The trade-off between yield and $WP_{C}$ for the irrigation treatments during the wet years clearly showed that higher and lower irrigation treatments were not optimum; however, 60% and 70% FC resulted in a win–win trade-off (**Figure 11c**). A clear response for the trade-off between yield and $WP_{C}$ during the dry years represents that an irrigation treatment lower than 50% FC lies in the lose–lose quadrant for the Mecosta location with loamy sand soil (**Figure 11d**). This trade-off analysis exhibited contrasting patterns for the wet and dry years across the irrigation treatments. During the dry and wet years, deficit irrigation (<40% FC) decreased the yield drastically in varying magnitudes, while moderate irrigation (40%–60% FC) remained optimum in terms of yield and $WP_{C}$ during these years. The irrigation treatments above 60% FC remained inefficient when compared with the standard (50% FC). In addition, these higher irrigation applications also increased the disease incidence in crops, ultimately affecting the yield and $WP_{C}$ . The yield loss in loamy sand soil was comparatively more prominent at 14%–32% for the lower irrigation treatments (rainfed and 20% and 30% FC), with a subsequent decrease in $WP_{C}$ by 10%–17% across all the years. Irrigation higher than 50% FC showed an increase in both yield and $WP_{C}$ through simulation, but may adversely affect the yield and $WP_{C}$ . Fang and Su (2019) and Evett et al. (2012) revealed that the soil strongly influences the $WP_{C}$ due to its structure and water-holding capacity. Better yield and $WP_{C}$ can be achieved under optimum irrigation (Bani-Hani et al., 2018), while over- and under-irrigation significantly influence the yield and $WP_{C}$ (Waqas et al., 2021; Akkamis and Caliskan, 2023).

#### 3.2.2 Correlation of irrigation events and development stages based on the irrigation regimes

**Figure 12:**
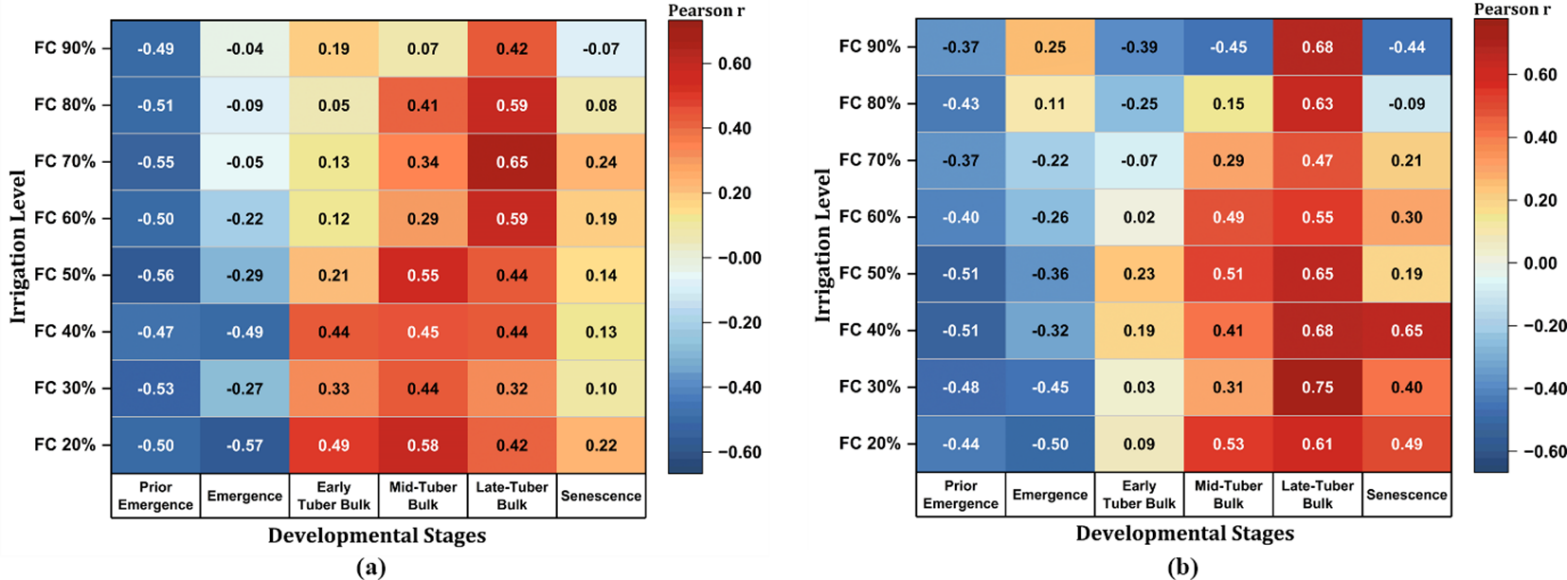
Heat map for Montcalm **(a)** and Mecosta **(b)** based on the Pearson’s correlation coefficients for the 2014–2024 growing seasons.

**Figure 12** presents a heat map of the Pearson’s correlation coefficients between the irrigation regimes and the different developmental stages of potato production for the 2014–2024 growing seasons. The analysis considered two different soil textures—sandy loam and loamy sand—under irrigation regimes ranging from 20% to 90% FC. The visualization showed positive (red) and negative (blue) correlations in terms of rainfall and irrigation provision at different developmental stages. At Montcalm, with a sandy loam soil, the negative correlations during the prior emergence and emergence stages suggest that the early-season rainfall was sufficient in reducing the need for additional irrigation. As growth progressed into the early-tuber bulking stage, the climate trend revealed a decline in rainfall, resulting in a positive correlation, highlighting the increased reliance on irrigation to meet the soil moisture requirements. The mid-tuber and late-tuber stages exhibited the greatest positive correlations, particularly for 40%–70% FC, coinciding with the peak temperatures and moderate rainfall trends. During the senescence stage, a slight increase in rainfall corresponded with weaker correlations, implying that irrigation beyond rainfall may not be necessary as the crop approaches maturity.

The findings for Mecosta, with a loamy sand soil, followed a pattern similar to that in Montcalm, but with some key differences. Early-season negative correlations at the prior emergence and emergence stages were present, although less pronounced. As rainfall declined during the early-tuber bulking stage, the correlation shifted to positive values, signifying increased irrigation events during these stages. The mid-tuber and late-tuber bulk stages maintained high positive correlations across multiple irrigation levels, particularly at 40% and 50% FC, emphasizing a greater dependence on irrigation to meet the soil moisture requirements. Notably, during the senescence stage at Mecosta, the positive correlation suggests that continued irrigation is crucial even in the later stages of potato production. Overall, the mid-tuber and late-tuber bulk stages emerged as the most critical phases for irrigation management. Both soil characteristics displayed peak correlations during these periods coinciding with the rising temperatures and the moderate but insufficient rainfall trends. Overall, while sandy loam soils benefit from moderate irrigation levels (40%–70% FC) during the bulk stages, which gradually decrease at senescence, loamy sand soils require consistent irrigation (40%–50% FC) that extends into the senescence stages of potato development.

## 4 Conclusions

The model successfully calibrated and validated the potato yield and $WP_{C}$ simulation using the collected field experiment data for the 50% and 70% FC irrigation treatments across sandy loam and loamy sand soils. The scenarios were further simulated for 10 years (2014–2024), with inter-annual seasonal variability in rainfall, with the years classified as wet (>312.9 mm), normal (256–312.9 mm), and dry (<256 mm). The statistical analysis of the model demonstrated satisfactory performance of the AquaCrop model in simulating the potato yield, as the model simulated the yield with ±10% accuracy and an IA of 0.999 for both irrigation treatments (50% and 70% FC). The observed variability in the yield simulations was attributed to the water availability, the temperature fluctuations, and the crop developmental stages, which in turn effected the canopy development, evapotranspiration, water use, and tuber formation. The SWC results simulated through the model under 50% and 70% FC were satisfactory, with IA and NSE closer to 1. The model performance may be influenced by uncertainties in the input data, the parameterization, and the complexity of crop response simulated under variable environmental conditions. Scenario analysis revealed that the soil type influenced the yield and $WP_{C}$ for all irrigation treatments. The trade-off analysis showed 40%–60% FC as the optimal irrigation treatment range, lying in the win–win scenario with comparatively higher yield and $WP_{C}$ , which outperformed the low irrigation scenarios (rainfed and 20% and 30% FC) and the over-irrigation scenarios (80% and 90% FC), taking into consideration all years and the different soil characteristics. The heat map of the Pearson’s correlation coefficients between the irrigation regimes and the different developmental stages of potato production, over the period of the scenario analysis, revealed the mid- to the late-tuber stages as the critical stages for irrigation supplementation across both soil characteristics. These findings highlight the importance of irrigation optimization in selecting the most effective approach at the most pertinent development stages of crop production for sustainable water use and increased $WP_{C}$ under changing climate scenarios.

## Data Availability

The raw data supporting the conclusions of this article will be made available by the authors, without undue reservation.
